# On a Case of Pyocyanic Meningitis, Treated and Healed by Means of Intraspinal Serumtherapy

**Published:** 1918-11

**Authors:** 


**On a Case of Pyocyanic Meningitis, Treated and Healed by Means of Intraspinal Serumtherapy.** By MM. J. ABADIE and GUY LAROCHE. *Bulletin de l'Académie de Médecine.* July, 2, 1918.

Pyocyanic meningitis proves extremely rare. The authors had the opportunity of studying a case of that disease, and relate it with many details. They believe it is, as yet, the first time that intraspinal autoserumtherapy has been resorted to in the treatment of that disease.

G. was wounded on November 11, 1916, by a large shell fragment fracturing the occipital; the dura-mater was severely injured, the brain substance issuing from the skull. The patient was trephined, and the projectile removed. On Nov. 20, began an abundant flowing out of spinal fluid. From December to January the patient's condition was as follows : alternative improvements for 2 or 3 days, followed by sudden aggravating for some hours, marked by temperature rising to 40.5° C., pulse at 140, torpor, blindness, dumbness.

By the end of November, the dressings soaked with spinal fluid were noticed to take spontaneously a bluish-green color after a few hours. A lumbar puncture was practised; the fluid thus punctured was put in culture tubes and a few strains of pyocyanic bacilli developed.

On January 5, the trial of intraspinal autoserumtherapy was decided upon. The presence of the specific anticorps in the patient's serum had been previously ascertained by agglutinating tests.

Bacilli obtained after a lumbar puncture, and grown in peptonised water, were agglutinated in 25 minutes at 1/800, in 6 hours at 1/1000. This test proved negative at 1/2000 and also at 1/100 with two normal control sera.

On January 8, 3 cc. of the patient's serum was injected intraspinally; his condition improved immediately. A second similar injection was practised a fortnight after; this was followed by a very rapid and as complete a recovery as possible, according to the severity of the injury of the skull.

